# Exposure to *Chlamydia trachomatis* Infection in Individuals Who Are Newly Diagnosed with HIV and Antiretroviral-Naïve from Belém, Northern Brazil

**DOI:** 10.3390/vaccines10101719

**Published:** 2022-10-14

**Authors:** Simone da Silva Góes, Ricardo Roberto de Souza Fonseca, Maria Eduarda de Sousa Avelino, Sandra Souza Lima, Misma Suely Gonçalves Araújo de Lima, Rogério Valois Laurentino, Maria Alice Freitas Queiroz, Felipe Bonfim Freitas, Antonio Carlos Rosário Vallinoto, Ricardo Ishak, Luiz Fernando Almeida Machado

**Affiliations:** 1 Biology of Infectious and Parasitic Agents Post-Graduate Program, Federal University of Pará, Belém 66075-110, PA, Brazil; 2 Virology Laboratory, Institute of Biological Sciences, Federal University of Pará, Belém 66075-110, PA, Brazil; 3 Evandro Chagas Institute, Health Ministry of Brazil, Ananindeua 67030-000, PA, Brazil

**Keywords:** *Chlamydia trachomatis*, HIV, co-infection, public health, vulnerability

## Abstract

*Chlamydia trachomatis* is one of the most prevalent sexually transmitted bacteria worldwide and may increase the risk of other sexually transmitted infections (STIs) including the human immunodeficiency virus (HIV). This study describes the seroprevalence of *C. trachomatis* infection among antiretroviral-naïve patients who are newly diagnosed with HIV in the city of Belém, Pará, in the Amazon region of Brazil. A cross-sectional study was carried out between January 2018 and January 2019 in 141 people living with HIV/AIDS (PLHA) who were followed up in a specialized unit of the public health network of Pará. The investigation of IgG antibodies against *C. trachomatis* was performed by enzyme immunoassay. Sociodemographic and sexual behavior information were obtained through a questionnaire. The prevalence of IgG anti-*C. trachomatis* antibodies was 64.8% (92/141). The majority of individuals were young, heterosexual, single men who did not use condoms during sexual intercourse and had no history of STIs. No significant differences were found when comparing any clinical or demographic data between groups. Our results demonstrated a high rate of exposure to *C. trachomatis* in newly diagnosed HIV-infected individuals in the Amazon region of Brazil, and all PLHA should be screened for *C. trachomatis* to decrease transmission of the bacteria and prevent the clinical manifestations of chronic infection.

## 1. Introduction

Chlamydia is the most prevalent bacterial sexually transmitted infection (STI) worldwide and is caused by *Chlamydia trachomatis* (CT) [1,2]. It is estimated that more than 127 million cases of infection occur annually worldwide [3]. Persistent infection can cause several diseases including pelvic inflammatory disease (PID), ectopic pregnancy and infertility in women, as well as urethritis and prostatitis in men, in addition to various other manifestations such as conjunctivitis and proctitis [4,5]. Most CT infections are asymptomatic and, even with well-established treatment, the lack of knowledge about the infection can facilitate the transmission of the bacteria and also favor its spread to extragenital sites, including the oropharynx and rectum [6].

STIs are among the most well-established risk factors for human immunodeficiency virus (HIV) infection, as they can facilitate HIV transmission by breaking through protective mucosal barriers and recruiting cells such as macrophages and CD4+ T lymphocytes to the site of infection [7,8].

Regular screening for *CT* infection is important and necessary to identify groups with higher prevalence and to establish control measures to prevent transmission of the infection, especially in PLHA, who may develop more severe symptoms [7]. Although *CT* infection is very common throughout the world, there are few studies on the prevalence of *CT* in people living with HIV/AIDS (PLHA). Recent studies show that the prevalence of *CT* in PLHA varies from 3.2% to 12.5% in populations from different countries [9,10,11]. In Brazil, the prevalence of CT infection in PLHA is still poorly understood. The most recent study was carried out in 2017, evaluated only women infected with HIV-1 and showed an average prevalence of 2.5% of *CT* infection [12].

The epidemiology of infectious diseases changes between different regions, constantly influenced by cultural, social, economic and environmental factors. Therefore, understanding the particularities involved in each population is essential to promote better control of infections [13]. Seroprevalence, based on the measurement of the presence of specific antibodies in a given population, can be used in addition to traditional and molecular methods of epidemiological surveillance of infections [14]. Serological assays have potential as epidemiological tools to quantify unmet needs, inform service planning, evaluate interventions, including screening and treatment, and are an important tool to assess *CT* and HIV co-infections [15,16].

In the northern region of Brazil, the largest in territorial extension, little is known about the prevalence of *CT* infection in PLHA, especially in people newly diagnosed with HIV infection. The present study aimed to describe the seroprevalence of IgG anti-*C. trachomatis* antibodies in individuals recently diagnosed with HIV from the city of Belém, Pará and the factors associated with the infection.

## 2. Materials and Methods

### 2.1. Type of Study and Ethical Aspects

The present study is cross-sectional and descriptive. The Research Ethics Committee with Human Beings of the Institute of Health Sciences of the Federal University of Pará, Brazil approved the study under protocol number 2.601.161. Written informed consent was obtained from all PLHA and a structured epidemiological questionnaire (through an interview) was applied.

### 2.2. Study Design

**Figure 1:**
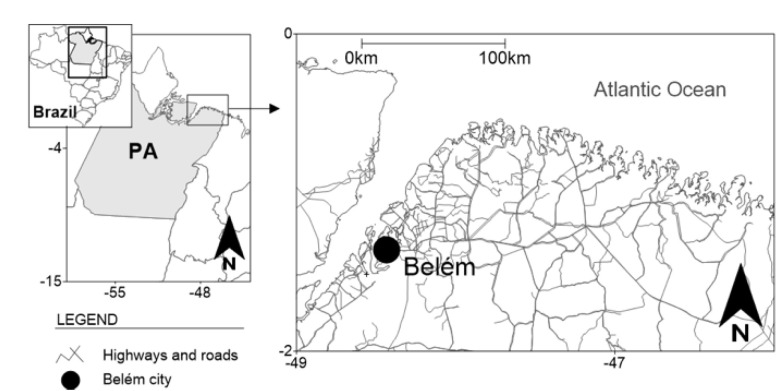
Geographic location of the city of Belém, state of Pará (PA), northern Brazil.

The study group included 141 newly diagnosed with HIV individuals, who spontaneously sought health assistance in the Centre for Health Care in Acquired Infectious Diseases (CASA DIA), which is a specialized public-health service that provides treatment for HIV/AIDS patients located in the city of Belém, capital of the state of Pará, northern Brazil (Figure 1) from January 2018 to January 2019. All study participants were antiretroviral-naïve or had not yet started highly active antiretroviral therapy (HAART).

The inclusion criteria were: older than 18 at collection time; were diagnosed with HIV infection for less than three months; agreed to participate in this study by signing the informed consent form; and answered the epidemiological questionnaire. The exclusion criteria were people with cognitive impairment who were unable to answer the questionnaire in an appropriate way.

Recruitment of study participants at CASA DIA was carried out on the day of the week when PLHA had their first clinical appointment after becoming aware of their HIV infection. During the waiting time, PLHA were informed of the study objectives and invited to participate. The subjects who agreed to participate in the research signed the consent form and provided information such as age, sex, marital status, schooling, monthly income, condom use in sexual practice, sexual intercourse with a sex worker and history of STIs, through a semi-structured questionnaire. The refusal rate to participate in the study was around 8.3%.

### 2.3. Sample Size

The determination of the sample size was based on the estimated prevalence of *CT* in women in Belém, Pará (11%) [13] and the estimated number of enrollments in CASA DIA in 2020 (approximately 812 new cases of HIV infection). An estimated 10% prevalence of *CT* infection in PLHA resulted in a minimum sample size of 140 individuals. 

### 2.4. Laboratory Tests

From each participant, a peripheral blood sample (5 mL) was collected by a vacuum collection system in a tube containing EDTA as anticoagulant. Plasma was separated by centrifugation (9000 rpm for 10 min) and stored at −20 °C until the moment of use, in the Laboratory of Virology, Institute of Biological Sciences, Federal University of Pará, where all of the laboratory tests were performed. Each sample was submitted to the enzyme immunoassay for the detection of *CT*-specific IgG antibodies (Serion ELISA Classic Chlamydia trachomatis IgG; Serion Diagnostics, Würzburg, Germany) according to the manufacturer’s instructions. The Serion ELISA Classic Chlamydia trachomatis IgG is a species-specific IgG antibody against *CT*-specific immunoassay, and according to the manufacturer’s instructions the cut-offs used in our laboratory for predicting CT blood infection and IgG titer were established in ≥1:64 to be considered CT-antibody positive [17,18,19].

### 2.5. Statistical Analysis

All statistical procedures were performed in SPSS 21.0 for Windows (SPSS Inc.,New York, USA). Descriptive analysis of the data was performed, with distribution of relative frequencies and later the data were categorized and grouped. Then, the chi-square and G test were performed, with a significance level of 5% (*p* < 0.05) and indicating presumptive differences in *CT*-positive versus *CT*-negative groups for each epidemiological parameter.

## 3. Results

The overall seroprevalence of anti-*C. trachomatis* IgG was 64.5% (91/141). Regarding the participants who had IgG, most were male (72.5%), heterosexual (49.4%), aged between 18 and 30 years (49.5%), single (79.1%), more than 8 years of study (79.1%), with an income greater than the minimum wage (75.8%), who used condoms occasionally (54.9%), with a partner in the last six months (95.6%) and without a history of STIs (58.2%). The analysis of the data distributed in dichotomous categories proved to be significant in the association of the presence of the disease with the educational level (*p* < 0.0135); however, for the other parameters, no statistically significance level was observed (Table 1).

## 4. Discussion

In the Northern region of Brazil, the largest in territorial extension, little is known about the prevalence of *CT* infection in PLHA. In this report, the prevalence of anti-*C. trachomatis* antibodies found in PLHA was 64.5%, and this percentage was very similar to that found in the only previous study carried out in PLHA in the city of Belém, Pará, where 64.2% were positive for IgG and 12.6% were positive for IgM [20]. This demonstrates a high rate of exposure to *CT* in this specific population and serves as an alert for greater screening of *CT* infection in the clinical follow-up of patients, which will favor adequate treatment and decrease the chain of transmission of *CT*.

However, the seroprevalence was much higher than that found in PLHA from Georgia (23.9%) [21]; in infertile women in Melbourne (37.0%) [22]; and in the general population of women with sub-fertility in Samoa (50.0%) [23]. Most recent studies in Brazil on the epidemiology of *CT* infection seek to detect the present/active infection, with the investigation of the presence of the bacteria through molecular techniques. In the Northern region, studies carried out in the state of Pará show a prevalence of 4% in young women from Marajó Island [24] and 11% in women from Belém, Pará [25]. The prevalence of *CT* infection in the state of Amazonas was 4.3% in women with HIV [13] and 12% in men with HIV [26].

When evaluating the data on the highest seroprevalence of *CT* in males, we found that the majority were between 18 and 30 years of age, were heterosexual and frequently had sex without condoms. The seroprevalence of anti-*C. trachomatis* antibodies was higher in men and the majority of patients who had antibodies were young, between 18 and 30 years of age, heterosexual and often had sex without a condom. The seroprevalence of anti-*C. trachomatis* antibodies found in the present study was much higher than that reported in HIV-negative women in Africa [27] and the United States of America [28]. According to Silva et al. [14], STIs increase the risk of HIV transmission and are related to more severe and earlier signs and symptoms in PLHA due to the co-infection system increasing the viral load in body secretions.

In the present study, we observed that HIV infection in individuals who were previously or concurrently infected with *CT* was associated with low TCD4+ and TCD8+ lymphocyte counts, and due to the absence of ART may have an increased risk of infection and transmission. Behavioral risk factors correlated with sex without condoms, reports of multiple partners and relationships with sex workers should be considered and monitored by health professionals in order to restrict the spread of STIs and HIV. Of the 141 patients evaluated, 40.42% (58/141) reported having STIs prior to the current co-infection condition; these findings reveal a possible association between STIs and HIV positivity with the aforementioned behavioral risk factors.

Additional data observed are that, of the 141 individuals in this study, most reported using condoms sometimes or not using them during sexual acts and all individuals reported understanding the fundamental role of condom use in the prevention and transmission of STIs. Our findings indicate that the model of current prevention and condom-use campaigns manage to convey the importance of protection; however, other approaches are needed to improve awareness of condom use.

Despite the relevant usefulness of serological tests for epidemiological screening of infections, it is important to consider possible cross-reactions with antibodies produced against other infectious agents. In the case of *CT*, cross-reactions against antibodies from other Chlamydia species may occur [29,30]. Therefore, it is important to associate molecular tests with serological screening to confirm *C. trachomatis* infection whenever possible.

## 5. Conclusions

In conclusion this study detected a high prevalence of IgG anti-*C. trachomatis* antibodies in PLHA in the city of Belém, Pará, northern Brazil. The relevant characteristics of seropositive PLHAs have been established and should serve for the establishment of control and prevention measures for *C. trachomatis* infections by local authorities to promote the health of the population in general and especially of PLHA.

## Figures and Tables

**Table 1:** Epidemiological and behavioral characteristics of PLHA with a concurrent or previous *C. trachomatis* infection from city of Belém, Pará, Brazil.

Characteristics	Total (*n* = 141)		CT IgG- (*n* = 50)		CT IgG+ (*n* = 91)		*p*-Value
	N	%	N	%	N	%	
**Sex**							
Male	107	75.9	41	82.0	66	72.5	0.2927 ^a^
Female	34	24.1	9	18.0	25	27.5	
**Sexual orientation**							
Homosexual	47	33.3	13	26.0	34	37.4	0.3165 ^a^
Heterosexual	73	51.8	28	56.0	45	49.5	
Bisexual	20	14.2	9	18.0	11	12.1	
Not informed	1	0.7	0	0.0	1	1.1	
**Age group (years)**							
18–30	73	51.8	28	56.0	45	49.5	0.4887 ^a^
31–40	37	26.2	10	20.0	27	29.7	
>40	30	21.3	11	22.0	19	20.9	
Not informed	1	0.7	1	2.0	0	0.0	
**Marital status**							
Single	110	78.0	38	76.0	72	79.1	0.8946 ^b^
Married	28	19.9	11	22.0	17	18.7	
Divorced/Widowed	3	2.1	1	2.0	2	2.2	
**Education level**							
Elementary school	35	24.8	16	32.0	19	20.9	0.0135 ^a^
High school	75	53.2	18	36.0	57	62.6	
College degree	30	21.3	15	30.0	15	16.5	
Not informed	1	0.7	1	2.0	0	0.0	
**Family income**							
Up to 1 minimum wage	30	21.3	11	22.0	19	20.9	0.8739 ^a^
More than 1 minimum wage	108	76.6	39	78.0	69	75.8	
Not informed	3	2.1	0	0.0	3	3.3	
**Condom use**							
Always	32	22.7	12	24.0	20	22.0	0.7464 ^a^
Never	27	19.1	8	16.0	19	20.9	
Sometimes	80	56.7	30	60.0	50	54.9	
Not informed	2	1.4	0	0.0	2	2.2	
**Sex with sex worker**							
Yes	26	18.4	8	16.0	18	19.8	0.8667 ^a^
No	106	75.2	37	74.0	69	75.8	
Not informed	9	6.4		0.0		0.0	
**STIs History** ^+^							
Yes	57	40.4	19	38.0	38	41.8	0.7982 ^a^
No	84	59.6	31	62.0	53	58.2	

^+^ Last 3 months. ^a^ Fisher’s exact test; ^b^ Chi-square test.

## Data Availability

All data referred to this study are available on the manuscript.
